# Piece of Evidence. Commentary: Ancestral Mental Number Lines: What Is the Evidence?

**DOI:** 10.3389/fpsyg.2016.00553

**Published:** 2016-04-22

**Authors:** Rosa Rugani, Giorgio Vallortigara, Konstantinos Priftis, Lucia Regolin

**Affiliations:** ^1^Department of General Psychology, University of PadovaPadova, Italy; ^2^Center for Mind/Brain Sciences, University of TrentoRovereto, Italy

**Keywords:** numerical cognition, number sense, domestic chick, spatial numerical associations, mental number line

In a recent comment, Núñez and Fias (2015) argued that our study (Rugani et al., 2015a) failed to demonstrate the existence of a linear numbers-space mapping. Defining the specific structure (straight vs. curve line, logarithmic vs. linear, 2D vs. 3D, hyperbolic or parabolic!) of chicks' (or newborn humans') number-space mapping was not the aim of our research (Rugani et al., 2015a). The aim of our study was, instead, to investigate the existence of spatial numerical associations in subjects without language or cultural experience (i.e., chicks), independently of the exact nature of this association. We found that 3-day-old domestic chicks associate small numbers with the left side of space and large numbers with the right one (Rugani et al., 2015a), excluding a role of language or culture on the original number-space association. Therefore, we claimed that number space mapping in chicks resembles the humans' Mental Number Line (MNL), a well-known description of the fact that adult humans associate small numbers with the left side and large numbers with the right side (Umiltà et al., 2009; Dehaene, 2011).

Núñez and Fias's rebut our results because they do not comply with a mathematical definition of linear mapping. A focus about literal definitions, however does not detract interest from the fact that evidence has been recently accumulating of the existence of language-independent associations of space and number (Rugani et al., 2007, 2010, 2011, 2014; de Hevia and Spelke, 2009, 2010; Lourenco and Longo, 2010; Adachi, 2014; Drucker and Brannon, 2014; Bulf et al., 2016).

Besides being overly concerned with the appropriateness of definitions, Núñez and Fias claim that our results could be explained in terms of chicks' asymmetric behavior. Indeed, several behavioral asymmetries have been demonstrated in chicks (Daisley et al., 2009; Rogers et al., 2013); in all these cases biases referred to well defined responses scored for one side consistently exceeding those scored for the opposite direction. Nonetheless, this was not our case because, in contrast with Nunez and Fias claim about our analyses, we showed that our chicks' performance was symmetrically distributed around a mean of 0.50 (i.e., chance level suggesting lack of asymmetry), and it was equally distributed across all conditions (small vs. large number trials) and across all experiments. We would like to encourage whoever is concerned (as well as Núñez and Fias) to directly re-compute the analyses from our dataset available online (Rugani et al., 2015a,b).

Núñez and Fias also commented that, because in our experiments chicks experienced a number during training and new numbers during testing, the novelty effect could be produced by an asymmetrical processing that could explain chicks' behavior. That is, given the logarithmic compression of the human MNL (but never demonstrated in chicks), large numbers are more difficult to be distinguished from the reference (less novelty), whereas small numbers are easier to be distinguished from the reference (more novelty). First, it should be noticed that responses to “novelty,” in the articles quoted by Núñez and Fias, were guided either by the left hemisphere (Vallortigara et al., 1996) or, by means of different paradigms, by the right hemisphere (Vallortigara and Andrew, 1991); thus, novelty processing cannot be systematically associated only with one hemisphere. Second, even if novelty were processed by the right hemisphere of our chicks, leading them to associate small numbers with the left side of space, there is no explanation why larger numbers would be associated with the right side of space, unless we hypothesize that in this case less novelty is processed by the left hemisphere! We suggest, instead, that the direction of bias that we found, even on the very first test and on the very first trial (Rugani et al., 2015b), was actually opposite depending on the initial training and as a function of number magnitude. For instance, when chicks were trained on five, initial bias to eight was to the right, whereas when chicks were trained on 20, the initial bias to eight was on the left.

In sum, the criticisms proposed by Núñez and Fias did not affect our results and their interpretation.

We believe that our study is a valid first step to better understand the phylogenetic and ontogenetic origin of spatial-numerical associations. Further studies are required to better understand the nature (e.g., logarithmic or linear) and the specific mapping rules that govern the MNL.

We are aware that we are just at the beginning of the investigation of number-space associations, but we strongly believe that future experimental studies will help to disentangle such a complex phenomenon.

## Author contributions

RR and KP wrote the manuscript. LR and GV provided critical revision. All authors approved the final version of the manuscript for submission.

### Conflict of interest statement

The authors declare that the research was conducted in the absence of any commercial or financial relationships that could be construed as a potential conflict of interest.
